# Modulation of the PLLA Morphology through Racemic Nucleation to Reach Functional Properties Required by 3D Printed Durable Applications

**DOI:** 10.3390/ma14216650

**Published:** 2021-11-04

**Authors:** Doina Dimonie, Silvia Mathe, Manuela Maria Iftime, Daniela Ionita, Roxana Trusca, Sorina Iftimie

**Affiliations:** 1 National Institute for Research and Development in Chemistry and Petrochemistry, 202 Splaiul Independentei, 060021 Bucharest, Romania; ddimonie@yahoo.com; 2 Doctoral School “Applied Chemistry and Materials Science”, Politehnica University of Bucharest, 1-7 Gheorghe Polizu, 011061 Bucharest, Romania; 3 “Petru Poni” Institute of Macromolecular Chemistry, 41A Grigore Ghica Voda Alley, 700487 Iasi, Romania; ciobanum@icmpp.ro (M.M.I.); dgheorghiu@icmpp.ro (D.I.); 4 Department of Science and Engineering of Oxide Materials and Nanomaterials, Politehnica University of Bucharest, 1-7 Gheorghe Polizu, 011061 Bucharest, Romania; truscaroxana@yahoo.com; 5 Department of Electricity and Magnetism, Solid-State Physics, and Biophysics, Faculty of Physics, University of Bucharest, 405 Atomistilor, 077125 Magurele, Romania; sorina.iftimie@fizica.unibuc.ro

**Keywords:** PLA, stereo-complexing, racemic nucleation, functional properties, filament, 3D printing

## Abstract

This paper presents an alternative for enhancing the durability of poly (L-lactide) (PLLA) by racemic nucleation following stereo-complexation with a selected poly (D-lactide) (PLDA). The compounds are obtained by melt blending of a PLLA grade, previously designed for 3D printing but with a low heat deflection temperature and impact resistance, with grades of PLDA differing in their molecular weight (Mw), D-lactide content (DS) and concentration. Our method considered how to reveal the racemic nucleation caused by stereo-complexation and its influence on functional properties. The FTIR study we performed showed that, depending on Mw, DS and concentration of the stereo-complexer (PDLA) used, bigger or smaller spectral changes can occur. The stereo-complexation was confirmed by the DSC analysis and, for the selected compound, by the POM, SEM, AFM microscopies, functional property and shapeability as 3D printing filaments. All the obtained results sustain the idea that, if a PLLA with M_w_ of 4.5 × 10^4^ g·mol^−1^ is modified with PDLA with a medium M_w_ of 11.6 × 10^4^ g·mol^−1^, medium DS of 4% and 1% concentration, a racemic nucleation is possible. It produces a racemic polylactic acid (PDLLA) with improved durability and good shapeability as 3D printing filaments. These results are explicable if the dependence of the intermolecular interactions appears between the PLLA and stereo-complexer PDLA. To enlarge the durable applicability of racemic polylactic acid (PDLLA), future research should identify other parameters controling the PLA stereo-complexing as the intensifying the mobility of the macromolecules, the finding of the optimal recemic cristalization window.

## 1. Introduction

Poly(lactic) acid (PLA) is a biodegradable, biocompatible, compostable, semi-crystalline, bio-based thermoplastic aliphatic polyester, which has a relatively low cost, outstanding strength and a high elastic modulus. Its good melt processability makes it a significant alternative to the petrochemical-based polymers such as polypropylene (PP), polystyrene (PS) and polyethylene (PE). [1,2,3,4]. Despite its many advantages, PLA is a brittle polymer with a low thermal deformation temperature, poor toughness and low crystallization rates [5,6,7,8,9,10]. These disadvantages limit its applicability, particularly for long-life (durable) items, including for the 3D printed grades designed for the automotive industry [11] which need key characteristics such as high temperature melt processability, significant toughness and durability [12]. Durability is defined as the property of polymeric materials to withstand environmental stresses during its life time so that the material’s performance not to be hindered [13]. Durability refers to those properties that can fail before the end of the item’s lifetime. These properties depend on the degradation stresses characterizing each application. For example, if temperature resistance is the main requirement for a certain application, then the thermal properties level will be the main expression of durability. If during its lifetime the plastic item works under continuous mechanical stresses, then the mechanical properties will define its durability [13]. To design the PLA-based material’s functional properties [14,15,16,17], including strong 3D printability and mainly considering polymer morphology [18], the following techniques are usually used: copolymerization [19], melt compounding with other polymers [20] and controlled crystallizations (in-mold annealing—under shear flow—through nucleation) [13,21,22,23,24,25,26]. Nucleation involves the formation, in a “controlled” manner and in the same volume unit, of more crystals with a smaller size, as in “spontaneous crystallization”, which generates the changing of the morphology. Obtaining a desired morphology in a “controlled” pattern improves physical, mechanical and optical properties, as well as dimensional stability, output at melt processing and the cost-performance index [27]. Nucleation can be achieved following different mechanisms, either by using nucleation/clarifying agents or by stereo-complexing (racemic nucleation) [13,27]. The nucleant is a solid salt that remains solid at the compounding temperature, and its particles represent the points around which the crystals develop. The higher the number of nucleation nuclei (particles of nucleant), the more new crystals appear and the smaller their size upon appearance [13].

The stereo-complexation is the result of selective interactions between two enantiomers, which have the same composition but different three-dimensional configurations [27]. The driving force of PLA stereo-complexation is the hydrogen bonds established between the -CH_3_ and O=C groups of the two enantiomers macromolecules. Following the formation of these secondary interactions, changes related to the adjacent functions to these groups also appear, namely the C-O-C or -C-O- linkages [28,29,30,31]. These interactions generate racemic nucleation with high rate and, therefore, the formation of a semi-crystalline morphology with small-sized crystallites represented by racemic or mixtures of racemic with a homopolymer type, spread into an amorphous matrix. [32,33,34,35,36] Compared to the parent polymers, the resulting racemic polylactic acid (PDLLA) has enhanced functional properties (mechanical strength, thermal and hydrolytic stability etc.) and therefore controlled durability [30,31,32,33,37,38].

The lactic acid contains an asymmetric optically active carbon, which produces two optical isomers—L-lactic acid (levo-rotatory) and D-lactic acid (dextro-rotatory) [35,36] and, consequently, the following enantiomers: poly (L-lactide) (PLLA), poly (D-lactide) (PDLA), meso poly (lactide) (meso-PLA) and racemic polylactic acid (PDLLA) [37]. The racemic nucleation of PLA in distinct conditions and blending ratios, by solution or melt blending, depends on its molecular weight (Mw) and D-lactide content (DS). It is known that the crystallinity decreases with the increase in the DS [39,40,41], but there are no data about the Mw influence or the optimal DS that favors stereo-complexation.

The goal of this paper is to find the proper molecular weight and D-lactide content of a PDLA that can be melt compounded with a PLLA to obtain a racemic polylactic acid with increased durability to be used as filaments for 3D printed items for long life applications in the automotive industry.

## 2. Materials and Methods

### 2.1. Experiments

**Figure 1:**
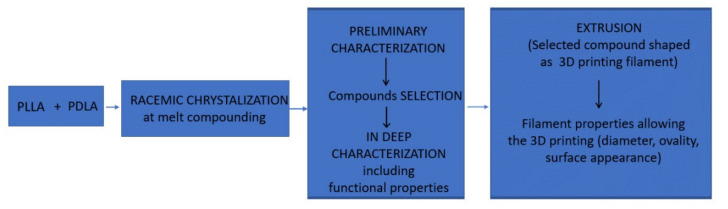
Complete study design for achieving racemized PLLA with functional properties required by 3D printing automotive applications.

In order to obtain PLLA for long-life 3D printed applications, the experimental program presented in Figure 1 was designed. Commercially available PLA grades with different molecular weights and D-lactide content (Table 1) were melt compounded using a common intensive melt-compounding rolling sequence (Brabender: 185 °C–200 °C, 100 rpm; laboratory roller: 100 °C–125 °C, 25 rpm) to obtain new compounds shaped as rolling sheets of 0.6 ± 0.05 mm thickness (composition as in Table 2).

### 2.2. Characterization

#### 2.2.1. Chemical Structure and Thermal Behavior

To find the influence of racemic complexation on chemical structure and thermal behavior, the sc-compounds were firstly characterized by Fourier transform-infrared spectroscopy in the attenuated total reflectance mode (FTIR-ATR) and then by differential scanning calorimetry (DSC). Each selected sc-compound was characterized in depth, mainly by studying its crystallization behavior (polarized optical microscopy—POM), morphology (scanning electron microscopy—SEM), surface appearance (atomic force microscopy—AFM), functional properties and shapeability as filaments for 3D printing.

For tracking the spectral changes characterizing the PLLA racemization, the following FTIR ranges have been studied: 3600–2800 cm^−1^, 1850–1700 cm^−1^, 1500–1000 cm^−1^ and 1000–700 cm^−1^ [42,43,44,45].

FTIR-ATR was carried out using Perkin Elmer Spectrum 100 equipment to record the FTIR spectra between 4000 and 650 cm^−1^. To obtain an optimal signal-to-noise ratio, 32 scans were performed. A clean, empty diamond crystal was used for the collection of the background spectrum.

To eliminate the sample’s thermal history [43,44], the DSC thermograms were recorded with 3 runs (2 heating and 1 cooling). The recording was performed on a DSC3 Mettler Toledo device, using the following procedure: first heating from 20 to 200 °C (10 °C/min), cooling from 200 to 20 °C (2 °C/min), second heating from 20 to 250 °C (10 °C/min) and a 2 min isotherm between each segment. The crystallinity (*X*) was calculated with the Equation (1), where the parameters represent: ${\Delta H}_{m}$—melting enthalpy, ${\Delta H}_{\mathrm{cc}}$—cold crystallization enthalpy, ${\Delta H}_{m}^{o}$—melting enthalpy of a 100% crystalline PLA (93.1 J·g^−1^) and $w_{PLA}$—mass fraction of PLA in the compound [44,45].

(1) $$\%\;X=\frac{{\Delta H}_{m}-{\Delta H}_{\mathrm{cc}}}{{\Delta H}_{m}^{o}\;\cdot \;w_{PLA}}\cdot \;100\%\;X=\frac{{\Delta H}_{m}-{\Delta H}_{\mathrm{cc}}}{{\Delta H}_{m}^{o}\;\cdot \;w_{PLA}}\cdot \;100$$

#### 2.2.2. Deep Characterization of Selected Blend

The selected sc-compound was characterized in depth, mainly by studying its crystallization behavior (polarized optical microscopy—POM, Leica Microsystems Inc., Morrisville, NC, USA, morphology (scanning electron microscopy—SEM (Tescan, Brno-Kohoutovice, Czech Republic), surface appearance (atomic force microscopy—AFM (A.P.E Research, Trieste, Italy), functional properties and shapeability as filaments for 3D printing.

The POM was performed with a Leica DM 2500M optical microscope (Leica Microsystems Inc., Morrisville, NC, USA) equipped with an objective of 10X, Mettler Toledo FP82HT heating plate and FP 90 central Processor Microscope (Mettler-Toledo, Columbus, OH, USA). The temperature program used was as follows: heating I: 10 °C/min; cooling I: 2 °C/min; heating II: 10 °C/min; cooling II: 2 °C/min. The second cooling included an isothermal temperature program maintained until complete crystallization, with the program established using the DSC results and at the temperature at which the crystallization began.

SEM micrographs were taken with an equipment Tescan Vega type, XMU model, for both samples’ transversal section and surface.

AFM analysis was conducted using an A.P.E Research equipment (A.P.E Research, Trieste, Italy), working in non-contact mode, on two scanning areas (3D) of 1 µm × 1 µm and 5 μm × 5 μm. The following surface properties were calculated: *mean square roughness* (radical of the standard deviation from a considered basic plane, using $RMS^{2}=\frac{1}{L}\int_{0}^{L}\left(z^{2}\right)dx$, where *L* is the length of the analyzed area and *z* is the standard deviation) and the *average roughness* (the arithmetic mean of the absolute values of the standard deviation from a considered basic plane ($R_{a}=\frac{1}{L}\int_{0}^{L}\lfloor zdx\rfloor$)) [46].

The subsequent functional properties were also measured: Izod impact resistance according to ISO 180/2019 and heat deflection temperature (HDT) matching the ISO 75-1/2020. The shapeability as filaments of the selected sc-PLLA was determined on a laboratory Gottfert extruder with a laboratory line for calibration, pulling and filament rolling (60 °C, 165–180 °C, 135 rpm).

## 3. Results

### 3.1. FTIR Analysis

The stereo-complexation was missing or was very small if the base-PLLA was stereo-complexed with PDLA with a high Mw (18 × 10^4^ g·mol^−1^) and medium DS (3.5%), or with PDLA with a high Mw (19 × 10^4^ g·mol^−^^1^) and high DS (12%) (Table 3).

**Figure 2:**
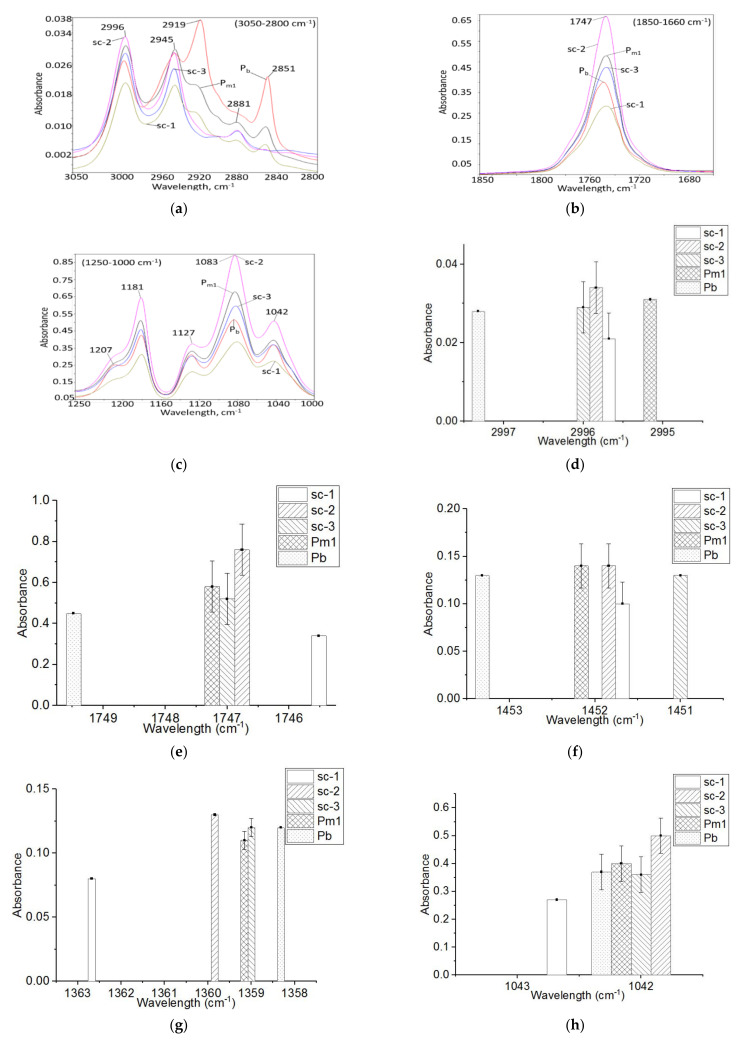
FTIR spectra of the sc-1, sc-2 and sc-3 compounds and those of the used PLA (base-PLLA and PDLA for stereo-complexing with medium M_w_ and medium D-lactide content). (**a**) 3050–2800 cm^−1^ range; (**b**) 1850 –1680 cm^−1^ range; (**c**) 1250–1000 cm^−1^ range; The absorbance at (**d**) 2995–2997 cm^−1^; (**e**) 1746–1749 cm^−1^ (**f**) 1451–1453 cm^−1^ (**g**) 1358–1363 cm^−1^; (**h**) 1042–1043 cm^−1^.

When the base-PLLA was compounded with PDLA with a medium molecular weight (11.6 × 10^4^ g·mol^−^^1^) and medium DS (4%) (sc-1 compound), the interactions appearing between the methyl (–CH_3_) and carbonyl (O=C) groups resulted from the spectra presented in Figure 2. By relating to the absorption of the same functional groups from the base-PLLA, the carbonyl absorption from 1747 cm^−1^ is 20% lower and its peak is 2 cm^−1^ shifted (Figure 2b,e). The –CH_3_ absorption from 2995 cm^−1^ is 25% smaller and its peak is 2 cm^−1^ shifted (Figure 2d). The –CH_3_ stretching from 1452 cm^−1^ is 23% lower (Figure 2f). The –CH_3_ absorption from 1360 cm^−1^ is 33.33% smaller and the peak is 4 cm^−1^ shifted (Figure 2g). The –CH_3_ stretching from 1042 cm^−1^ is 25% lower and the peak is 1cm^−1^ shifted (Figure 2h).

**Figure 3:**
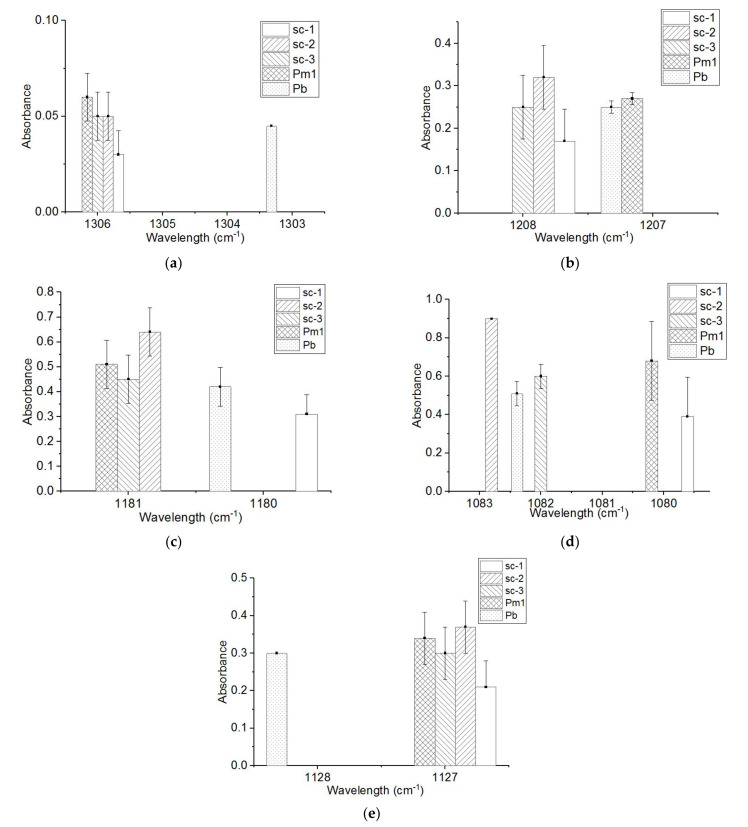
The FTIR absorptions in the 1128 cm^−1^–1306 cm^−1^ of the sc-1, sc-2 and sc-3 compounds; base-PLLA (Pb); and stereo-complexer PDLA with medium M_w_ and medium DS (Pm1)). (**a**) 1303–1306 cm^−1^; (**b**) 120–1208 cm^−1^; (**c**) 1180–1181 cm^−1^; (**d**) 1080–1083 cm^−1^; (**e**) 1127–1128 cm^−1^.

Spectral changes were also observed regarding the bonds adjacent to the –CH_3_ and –CO groups, namely the –C–O–C and –C–O– links (Figure 3). Additionally, the absorption from 1207 cm^−1^, assigned with the coupling of C–O–C stretching and CH_3_ rocking, is 32% smaller and shifted with 1 cm^−1^ (Figure 3b). The absorptions assigned with the C–O–C symmetric stretching also had the following changes: those from 1188 cm^−1^ are 26% smaller (Figure 3c), the one from 1081 cm^−1^ is 24% lower (Figure 3d), and the absorptions from 1058 cm^−1^ no longer exist. The absorption indicating the –C–O– stretching at 1127 cm^−1^ is 30% smaller (Figure 3e) and those from 1175 cm^−1^, assigned with the C–O–C asymmetric stretching, are not present.

### 3.2. DSC Analysis

**Figure 4:**
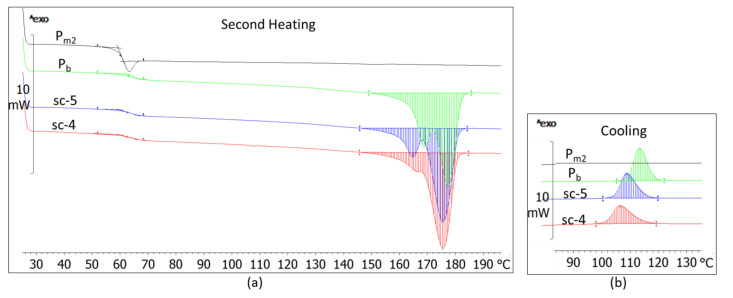
DSC thermograms of the sc-4 and sc-5 compounds and those of the blended polymers (Pb, P_m2_) after the second heating (10 °C/min) (**a**) and the cooling (2 °C/min) (**b**).

**Figure 5:**
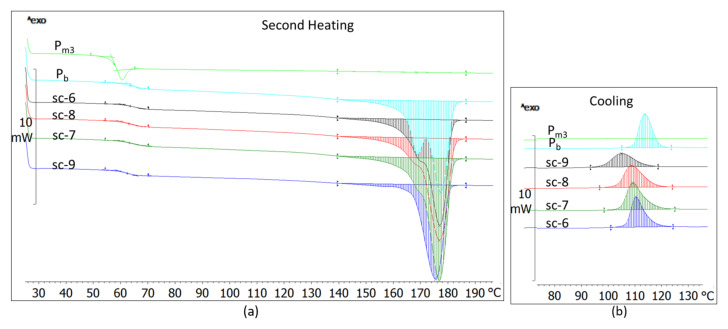
DSC thermograms of the sc-6, sc-7, sc-8 and sc-9 compounds and those of the compounded PLA grades (P_b_, Pm_3_) after second heating (10 °C/min) (**a**) and after cooling (2 °C/min) (**b**).

The compounds achieved through stereo-complexing the base-PLLA with PDLA with a high M_w_ (18.04 × 10^4^ g·mol^−1^) and medium DS (3.5%) have a glass transition lower with 1–3℃ as the base-PLLA (Figure 4, Table 4). Compared to the base polymer, these compounds have bimodal melting, which takes place in wider ranges moving to lower temperatures, and they have a 10–20% lower melting enthalpy (Figure 4a, Table 4). They also have a smaller crystallinity, crystallize in a wider range, shift to lower temperatures and have crystallization peaks at a couple °C lower than the base-PLLA (Figure 4b, Table 4). The same results were obtained in the case of stereo-complexing of base-PLLA with PDLA with high M_w_ (19 × 10^4^ g·mol^−1^) and high DS (12%) (Figure 5, Table 5).

These result show that if the base-PLLA is modified with PDLA with a high Mw and medium DS, or with a high Mw and high DS PDLA, then the morphological order of the resulting blends is significantly decreased. Considering the complete lack of stereo-complexing interactions between the base-PLLA and these two PDLA grades, proved by the FTIR spectra, the decreased morphological order of their compounds is explainable.

**Figure 6:**
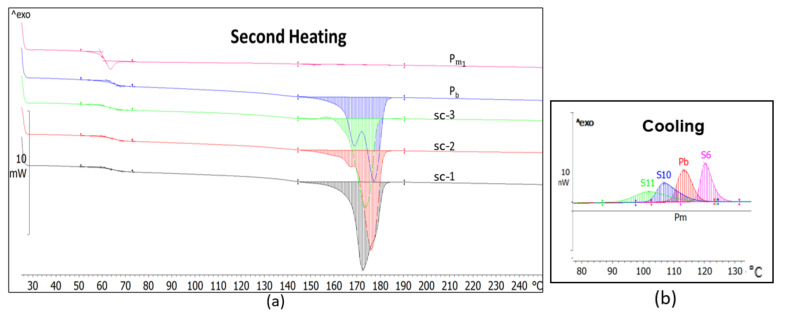
DSC thermograms of the sc-1, sc-2 and sc-3 compounds and of the blended PLA grades (P_b_, Pm_1_) after the second heating (10 °C/min) (**a**) and cooling (2 °C/min) (**b**).

**Figure 7:**
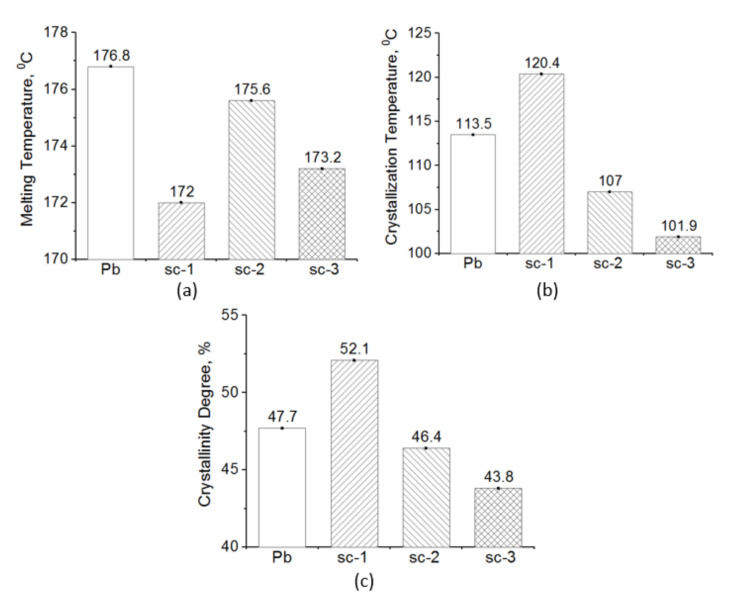
Thermal properties of the sc-1, sc-2 and sc-3 compounds vs. base-PLLA: melting temperature (**a**), crystallization temperature (**b**) and crystallization degree (**c**).

Unlike the compounds described above, those achieved by stereo-complexing the base-PLLA with PDLA with a medium M_w_ (11.6 × 10^4^ g·mol^−1^) and medium DS (4%) have different thermal behavior (Figure 6 and Figure 7, Table 6). The sc-1 compound has a glass transition almost similar to that of the base-polymer (Table 6) and melts in a mono-modal homogenous way unlike the bi-modal melting of the base-PLLA (Figure 6a and Figure 7a). The crystallization occurs narrowly and highly with a 7–12 °C temperature range as the base-PLLA, which means that the crystallites are smaller and regular in size, and they do not contain or have very few defects [47]. The compounds’ crystallinity is almost 7% greater considering the base-PLLA, the crystallinity, how the crystallization enthalpy increased by almost 6 J·g^−1^ (Table 6) and the crystallization’s 6–10% temperature increase (Figure 6b and Figure 7b, Table 6).

If in the case of the sc-1 compound—which contains small amount of stereo-complexer—a certain morphological ordering effect was found, the same was not observed with a larger quantity. As the crystallinity and the crystallization temperature values revealed, the thermal behavior of the sc-2 and sc-3 compounds demonstrates a relative decrease in the ordering morphological level (Figure 7, Table 6).

### 3.3. POM, SEM and AFM Morphologies

**Figure 8:**
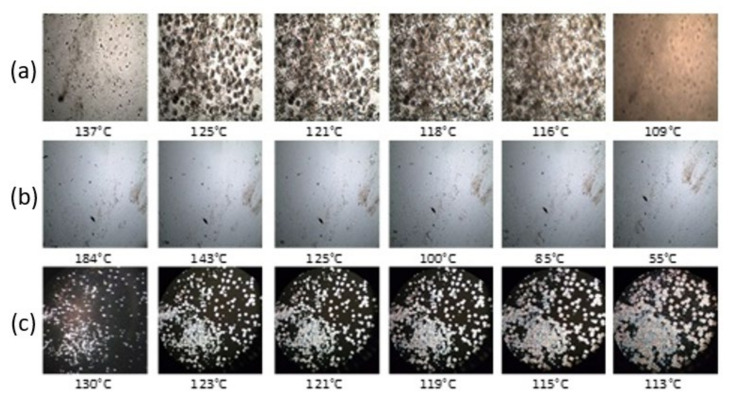
POM micrographs of (a) base-PLLA (P_b_) (**a**), stereo-complexer PDLA(P_m1_) (**b**) and resulting stereo-complexed compound sc-1 (**c**), taken during non-isothermal crystallization.

**Figure 9:**
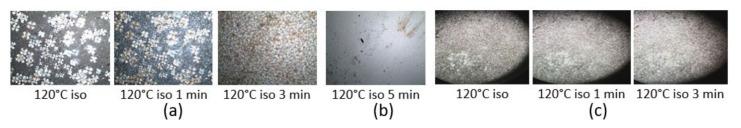
POM micrographs taken during isothermal crystallization, at 120 °C, of base-PLLA (P_b_) (**a**), stereo-complexer PDLA (P_m1_) (**b**) and resulting stereo-complexed compound sc-1 (**c**).

**Figure 10:**
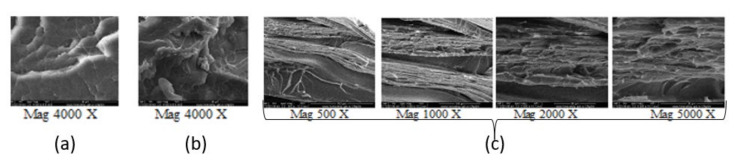
SEM micrographs base-PLLA (P_b_) (**a**), stereo-complexer PDLA (P_m1_) (**b**) and resulting stereo-complexed compound sc-1 (**c**).

The study of crystallization in non-isothermal conditions confirms the stereo-complexation of the compound coded as sc-1. Compared to the base-PLLA, this compound contains more small crystals in the same volume (Figure 8). The growth of a greater number of crystallites, in the same volume, was possible due to the increasing crystallization rate following the racemic nucleation after the stereo-complexation [48,49]. The crystallization in isothermal conditions reveals the same conclusion: the racemic nucleation followed the stereo-complexing because, at the same time and volume, fewer and larger small crystals appeared in the case of the base-PLLA, compared to numerous and smaller crystals for the sc-1 compound (Figure 9). According to the SEM micrographs, the stereo-complexed compound has a more ordered morphology (Figure 10). Unlike the two compounded polymers, as demonstrated by SEM micrographs, the sc-1 blend showed crystals arranging in lamellar structures at melt processing, probably along the direction of the shear stress action.

As shown by the parameters describing the surface roughness measured in two different areas (3D), the selected sc-1 compound has a smoother surface than that of the used PLA grades, because the roughness parameters have lower values (Table 7).

### 3.4. Functional Properties of Stereo-Complexed PLA

The functional properties of the selected compound sc-1 confirmed the idea that if the base-PLLA (M_w_ of 4.5 × 10^4^ g·mol^−1^) is modified with a PLA with medium molecular weight (11.6 × 10^4^ g·mol^−1^) and medium D-lactide content (4%), the measured functional properties are thus improved: the Izod impact resistance is 1.8 kJ/m^2^ higher and the HDT is 17 °C higher than the values characterizing the base-PLLA (Table 8).

### 3.5. Shapeability of Selected Stereo-Complexed PLA as Filaments for 3D Printing

**Figure 11:**
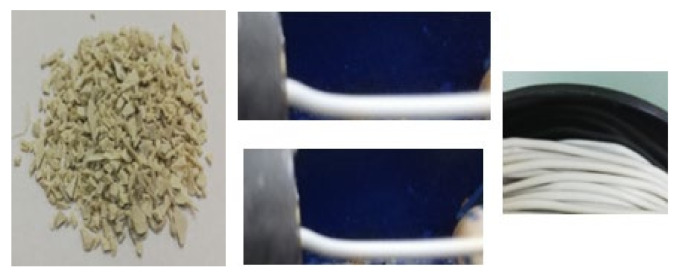
Shaping of selected racemic nucleated selected compound into 3D printing filaments.

The selected racemic-nucleated compound (sc-1) was shaped, with good results, as filaments for 3D printing (Figure 11). These filaments had both their diameter (variation from 1.90 to 1.80 mm) and their ovality in the allowed range (variation from 0.005 to 0.06 mm), had smooth surfaces and no defects, and they behaved accordingly when 3D printed.

## 4. Discussion

All of the obtained results reveal the dependence of the racemic nucleation on the molecular weight of the PDLA macromolecules, their DS content and the concentration of the stereo-complexer.

It is well known that relatively short macromolecules crystalize faster than the longer ones because the crystallization is diminished by their entanglement degree [50]. A medium or high DS content level can reduce the compound crystallinity because, with a great number of racemic connections between the macromolecules, the chain fragments that can be ordered by crystallization decrease. The bimodal melting observed in the case of the compounds that did not show racemic crystallization happened because during their cooling, at least two different-sized crystals appeared [51].

The racemic crystallization also depends on the concentration of the used PDLA. High concentrations do not favor racemic crystallization, because higher concentrations mean many entanglement points and, hence, few chain segments that can crystallize.

The thermal behavior of the selected sc-1 compound sustains the idea that racemic nucleation is possible if a base-PLLA with a M_w_ of 4.5 × 10^4^ g·mol^−1^ is modified with a PDLA with a medium M_w_ of 11.6 × 10^4^ g·mol^−1^ and medium DS of 4%. Consequently, the stereo-complexing effect depends not only on the length of the PDLA macromolecules and their D-lactide content but also on the concentration of the stereo-complexation modifier. All the DSC results sustain the conclusions of the FTIR study.

A reduction in roughness is proof that the selected compound has no phase separation, is homogenous and was not degraded at melt processing [52,53]. This degradation could cause surface micro-cracking and increase surface roughness [53]. These results also confirm the stereo-complexation of the two PLA grades, and, thus, their racemic nucleation—demonstrated by POM as well as SEM— differs fundamentally from the morphologies of the melt-compounded PLA grades.

Due to the more plentiful but smaller crystallites dispersing into the amorphous matrix represented by entangled macromolecules of the stereo-complexer PDLA (11.6 × 10^4^ g·mol^−1^ and DS of 4%), the resulted more ordered semi-crystalline morphology of the selected compound seems to be much more elastic than the base-PLLA and, therefore, to need higher broken energy. The values of the functional properties prove the improvement in the thermal and mechanical behavior of the base-PLLA, with it obtaining a grade with good shapeability as 3D printing filaments. To have proper 3D printing behavior, a polymeric material must melt and crystallize in narrow temperature ranges. Melting in a narrow range means uniform flow in the molten state during shaping of selected compounds into 3D printing filament. The crystallization describes the effect of cooling on the newly achieved 3D printed items, as well as on its removal from the 3D printing support [13,54,55,56]. As was shown in Section 3.2, the selected compound meets these conditions.

Compared to the current PLA filaments on the market, those attainable by racemic crystallized PLLA have improved durability because of the following advantages: controllable 3D printing behavior and the 3D printed items no longer softening in hot summer weather and no longer breaking at the smallest stresses acting on them. The developed filaments will be used for 3D printing in the automotive industry.

The composting behavior of the racemized PLLA will not be influenced by the presence of PDLA, because, through racemization, it interacted with PLLA and lost its individual properties. It is obvious that through racemic nucleation as a result of stereo-complexing, the functional properties and durability of PLA can be controlled. However, it is still necessary to identify other parameters that control the effect of stereo-complexation so that the advantages obtained can be enlarged. To better control stereo-complexing and thus racemic crystallization, future studies should increase the mobility of the PLA macromolecules involved in stereo-complexing so that the intensity of racemic interactions is higher. Further studies will also be performed to identify the racemic crystallization window, which represents the conditions in which racemic crystallization has the most convenient intensity

This study’s results showing an increase in PLA durability by racemic nucleation following stereo-complexing, and other results regarding the control of PLA crystallization by using nucleating agents [13], are part of a broader research agenda to increase the sustainability of PLA designed for 3D printing car parts.

## 5. Conclusions

The possibility of improving the functional properties of PLA by stereo-complexing of a PLA grade designed for 3D printing was studied. This PLA was melt compounded with different PLA grades, which differ in the length of the macromolecules, D-lactide content and concentration. The resulting compounds were characterized while considering structural methods (FTIR, DSC, POM, SEM and AFM) and procedures for identifying the functional properties and the shapeability of the selected compound as filaments for 3D printing.

All the obtained results sustain the idea that if PLLA with a M_w_ of 4.5 × 10^4^ g·mol^−1^ is modified with PDLA with a medium M_w_ of 11.6 × 10^4^ g·mol^−1^, medium DS of 4% and 1% concentration, then a racemic nucleation is possible, which would improve the functional properties

The obtained results can be explained by considering mainly the dependence of the macromolecules’ interactions on their molecular weight and D-lactide content. The relatively short polymer chains form crystals more readily than long chains, because the crystallization is diminished by the entanglement degree of the long chains. A medium or high content D-lactide can decrease the compound crystallinity, because at a large number of racemic connections between the macromolecules, the chain fragments that can be ordered by crystallization decrease. A higher concentration of modification PDLA initiates too many racemic nucleation points, and so the stereo-complexation becomes uncontrollable.

The filaments attainable by racemic crystallization are more durable as those existing in this time on the market because of the following improved properties: better 3D printability, without softening in hot summer and breaking at regular mechanical stresses.

## Figures and Tables

**Table 1:** Characteristics of the PLA grades used for stereo-complexation.

PLA * Grade	Function	Code	M_w_, g·mol^−^^1^	D-Lactide Content, %
PLLA D 850 ^2^*	Base-PLLA	P_b_	4.5 × 10^4/^Low	0.5/Low
PDLA D 3052	Stereo-complexer	P_m1_	11.6 × 10^4/^Middle	4/Middle
PDLA D 2003	Stereo-complexer	P_m2_	18.04 × 10^4/^High	3.5/Middle
PDLA D 4060	Stereo-complexer	P_m3_	19 × 10^4/^High	12/High

* INGEO of Natura Works; ^2^* Designed for 3D printing.

**Table 2:** The composition of the new stereo-complexed compounds (sc-compounds).

Codes	Blending Proportion for Getting Racemic Compounds, %								
	M* M_w_ and M*DS*			H* M_w_ and M*DS*		H* M_w_ and H*DS*			
	sc-1	sc-2	sc-3	sc-4	sc-5	sc-6	sc-7	sc-8	sc-9
P_b_	95.24	80	66.66	80	66.66	99	95.24	90.91	80
P_m1_	4.76	20	33.33	-	-	-	-	-	-
P_m2_	-	-	-	20	33.33	-	-	-	-
P_m3_	-	-	-	-	-	1	4.76	9.09	20

M*—medium; H*—high; DS*—D-lactide content.

**Table 3:** The absorbance of the compounds resulting from stereo-complexing of base-PLLA with PDLA with a high M_w_ (sc-4, sc-5, Pb, Pm2), and a high Mw and high DS (sc-6, sc-7, sc-8, sc-9, Pb, Pm3) (Mw and DS according to Table 1 and blends compositions as in Table 2).

FTIR Range	Wavelength, cm^−1^	Absorbance	Absorbance, H* M_w_ and M*DS*			Absorbance, H* M_w_ and H*DS*				
		P_b_	sc-4	sc-5	P_m2_	sc-6	sc-7	sc-8	sc-9	P_m3_
3600–2800 cm^−1^	2996	0.028	0.036	0.028	0.033	0.015	0.025	0.033	0.012	0.025
	2945	0.029	0.035	-	0.034	0.016	0.031	0.035	0.011	0.027
	2851	0.025	0.01	0.035	0.018	0.007	0.02	0.01	-	0.01
1850–1660 cm^−1^	1747	0.45	0.68	0.46	0.6	0.22	0.37	0.65	0.14	0.45
1480–1300 cm^−1^	1452	0.13	0.16	0.13	0.15	0.08	0.11	0.15	0.05	0.11
	1364	0.12	0.125	0.11	0.13	0.05	0.085	0.13	0.04	0.08
	1306	0.045	0.065	0.055	0.07	-	0.06	0.05	-	0.065
1275–1000 cm^−1^	1180	0.42	0.6	0.48	0.55	0.20	0.375	0.60	0.13	0.39
	1081	0.51	0.83	0.61	0.78	0.23	0.43	0.82	0.16	0.49
	1042	0.37	0.48	0.38	0.48	0.19	0.31	0.45	0.12	0.32

M*—medium; H*—high; DS*—D-lactide content.

**Table 4:** DSC behavior of the sc-4 and sc-5 compounds and those of the blended polymers (P_b_ and P_m2_).

Sample	DSC	Glass Trans.	Crystallization (exo)			Melting (endo)			Cryst.
		t_g_, °C	t_c_, °C	ΔHc, J·g^−1^	Range, °C	t_m_, °C	ΔHm, J·g^−1^	Range, °C	%
P_b_	Cooling	-	113.5	38.4	125–105	-	-	-	-
	Heating 2	65.7	-	-		176.8	44.3	144–186	47.7
P_m2_	Cooling	-	-	-	-	-	-	-	-
	Heating 2	61.5	-	-	-	-	-	-	-
sc-4	Cooling	-	106.6	32.2	120–98	-	-	-	-
	Heating 2	53.6	-	-	-	175	38.1	146–185	40.9
sc-5	Cooling	-	108.9	34.1	121–100	-	-	-	-
	Heating 2	63.6	-	-		175	35.5	146–185	38.2

**Table 5:** DSC behavior of the sc-6, sc-7, sc-8 and sc-9 compounds and of the blended PLA grades (P_b_ and P_m3_).

Sample	DSC	Glass Trans.	Crystallization (exo)			Melting (endo)			Cryst.
		t_g_, °C	t_c_, °C	ΔHc, J·g^−1^	Range, °C	t_m_, °C	ΔHm, J·g^−1^	Range, °C	%
P_b_	Cooling	-	113.5	38.4	125–105	-	-	-	-
	Heating 2	65.7	-	-		176.8	44.3	144–186	47.7
P_m3_	Cooling	-	-	-	-	-	-	-	-
	Heating 2	58.9	-	-	-	-	-	-	-
sc-6	Cooling	-	110.1	36.3	122–100	-	-	-	-
	Heating 2	64.7	-	-	-	176.5	47.8	144–186	51.4
sc-7	Cooling	-	108.9	35.8	122–98	-	-	-	-
	Heating 2	64.1	-	-	-	176.2	48.2	144–186	51.9
sc-8	Cooling	-	108.5	35.2	122–96	-	-	-	-
	Heating 2	63.4	-	-	-	176.3	44.5	144–186	47.9
sc-9	Cooling	-	104.7	31.2	118–92	-	-	-	-
	Heating 2	62	-	-	-	175	38.9	144–186	41.8

**Table 6:** DSC behavior of the sc-1, sc-2 and sc-3 compounds and of the blended polymers (P_b_ and P_m1_).

Sample	DSC	Glass Trans.	Crystallization (exo)			Melting (endo)			Cryst.
		t_g_, °C	t_c_, °C	ΔHc, J·g^−1^	Range, °C	t_m_, °C	ΔHm, J·g^−1^	Range, °C	%
P_b_	Cooling	-	113.5	38.4	125–105	-	-	-	-
	Heating 2	65.7	-	-	-	176.8	44.3	144–186	47.7
P_m1_	Cooling	-	-	-	-	-	-	-	-
	Heating 2	61.7	-	-	-	-	-	-	-
sc-1	Cooling	-	120.4	44.2	130–113	-	-	-	-
	Heating 2	64.4	-	-	-	172	48.4	144–186	52.1
sc-2	Cooling	-	107	35	122–96	-	-	-	-
	Heating 2	60.3	-	-	-	175.6	43.1	144–186	46.4
sc-3	Cooling	-	101.9	31	122–88	-	-	-	-
	Heating 2	63.1	-	-	-	173.2	40.8	144–186	43.8

**Table 7:** The parameters, mean square roughness (RMS) and mean roughness (Ra) characterizing the roughness of the selected sc-1 compound surface.

Sample/ Roughness Parameters	Scanning Area (3D), 1 µm × 1 µm		Scanning Area (3D), 5 µm × 5 µm	
	RMS (nm)	R_a_ (nm)	RMS (nm)	R_a_ (nm)
P_b_	4	3	10	7
P_m1_	2	2	6	5
sc-1	3	2	16	12

**Table 8:** Functional properties of base-PLLA and racemic nucleated PLA.

Specimen\Properties	Functional Property	
	Izod Impact Resistance (kJ/m^2^)	HDT (°C)
Base-PLLA	0.56	78
Stereo-complexed PLA (sc-1)	2.36	95

## Data Availability

The data presented in this study are available on request from the corresponding author.
